# Preparation and Characterization of Magnetite Talc (Fe_3_O_4_@Talc) Nanocomposite as an Effective Adsorbent for Cr(VI) and Alizarin Red S Dye

**DOI:** 10.3390/ma15093401

**Published:** 2022-05-09

**Authors:** AbdElAziz A. Nayl, Ahmed I. Abd-Elhamid, Ismail M. Ahmed, Stefan Bräse

**Affiliations:** 1 Department of Chemistry, College of Science, Jouf University, Sakaka 72341, Al Jouf, Saudi Arabia; ismadwy@yahoo.ca; 2 Nanotechnology and Composite Materials Research Department, Advanced Technology and New Materials Research Institute (ATNMRI), City of Scientific Research and Technological Applications (SRTA-City), New Borg Al-Arab, Alexandria 21934, Egypt; ahm_ch_ibr@yahoo.com; 3 Institute of Organic Chemistry (IOC), Karlsruhe Institute of Technology (KIT), Fritz-Haber-Weg 6, 76133 Karlsruhe, Germany; 4 Institute of Biological and Chemical Systems—Functional Molecular Systems (IBCS-FMS), Karlsruhe Institute of Technology (KIT), Hermann-von-Helmholtz-Platz 1, 76344 Eggenstein-Leopoldshafen, Germany

**Keywords:** impregnated Talc, Fe_3_O_4_, Cr(VI), Alizarin Red S, magnetic nanoparticles

## Abstract

In this work, the adsorption of Cr(VI) ions and the organic dye Alizarin Red S (ARS) was investigated using magnetite talc (Fe_3_O_4_@Talc) nanocomposite. Different characterization techniques such as scanning electron microscopy (SEM), Fourier transform infrared (FTIR) spectroscopy, X-ray Diffraction (XRD), and thermogravimetric analysis (TGA) were used to demonstrate the physical and chemical properties of the fabricated Fe_3_O_4_@Talc nanocomposite. In addition, the adsorption isothermic, kinetic, and thermodynamic properties were illustrated. The results demonstrate that the investigated adsorption processes obeyed the Langmuir isotherm model for Cr(VI) and the Freundlich isotherm model for ARS dye, with a maximum adsorption capacity of 13.5 and 11.76 mg·g^−1^, respectively, controlled by pseudo second-order kinetics. Regeneration and reusability studies demonstrated that the prepared Fe_3_O_4_@Talc nanocomposite is a promising and stable adsorbent with considerable reusability potential.

## 1. Introduction

With dramatic industrial and technological development large amounts of wastewater contaminated by dyes, toxic metals ions, and other compounds has been discharged into the environment as effluent. Such serious contaminants are poisonous, dangerous, and do not undergo degradation, causing harmful effects and threats to all aquatic life [1,2,3,4]. Contamination of water by such toxic wastes resulting from various industries (agrochemical residue/organic and/or inorganic pollutants) are highly hazardous and toxic to aquatic life, agriculture, and animal and human health [5]. Alizarin Red S (ARS) dye and hexavalent chromium (Cr(VI)) are examples of such extremely harmful environmental pollutants. ARS (C_14_H_7_NaO_7_S·H_2_O) dye is a derivative of alizarin, an example of anionic dyes, and a carcinogenic material [6]. It has a considerable resistance to biodegradation and destruction under normal conditions such as heat and light due to its complex structure of aromatic rings. Inhalation, swallowing or absorbance of ARS leads to very dangerous effects and can damage or irritate the lungs, mucous membranes, eyes, skin, and respiratory tract. This dye is a recalcitrant and durable contaminant; therefore, considerable efforts of scientists have been focused on the design and development of an effective, simple, and economical decontamination process [5,7]. Cr(VI) compounds are widely applied in various industrial processes such as electroplating and metal finishing, metallurgy, refractories, textile and pigment manufacturing, etc. They are considered one of the most common heavy metal ions, and have received great attention due to the dangerous threats they pose to natural ecosystems [8] and their high mobility and solubility in aqueous environments. Cr(VI) has highly toxic effects and can cause chronic toxicity, mutagenicity, and carcinogenicity [9,10]. Therefore, many countries and organizations have issued regulations and rules to minimize the serious effects of Cr(VI). According to the World Health Organization (WHO), the mandated maximum permitted concentration level is below 50 ppb and 100 ppb Cr(VI) for potable and surface waters, respectively [11,12,13,14]. Consequently, the reduction of these pollutants from industrial wastewater is becoming a crucial goal in environmental protection in order to eliminate this serious health and environmental hazard. The development of economic and eco-friendly materials to rapidly and efficiently dispose of such contaminants has attracted a great deal of attention among scientists. Currently, several physical, chemical, biological, and electrochemical treatment technologies are being studied to treat wastewater or other sources of water before use. These technologies include coagulation, precipitation, adsorption, membrane separation, nanofiltration, electrochemical, precipitation, coagulation, ozonation, and other techniques [15,16,17,18]. Among these technologies, adsorption methods are recognized to be one of the most competitive and effective technique for eliminating large amounts of toxic inorganic and organic substances thanks to their wide adaptability, cost-effectiveness, and eco-friendly operating systems [16,17,18,19,20,21,22,23]. Recently, the utilization of novel adsorbent materials with promising properties has been gaining great attention worldwide. Magnetic nanomaterials are among the most effective adsorbents, and can be utilized to remove all types of contaminations from water and wastewater [24,25,26]. To date, many different materials have been investigated to develop new adsorbents with promising properties for treating wastewaters. Among these natural and synthetic materials, zeolites and clay minerals have gained great attention due to their low cost, abundance in nature, excellent adsorptive characteristics, and chemical inactivity [27,28,29,30,31,32,33]. Currently, several potential traditional and nontraditional versions of these cost-effective and efficient adsorbent nanomaterials have been used to remove specific organic and inorganic pollutants from wastewater. Many of these adsorbent nanomaterials, such as magnetite talc (Fe_3_O_4_@Talc) nanocomposite, have the capability of capturing pollutants from aqueous solutions [16,24,34]. Up until now, research reports on the removal characteristics of Cr(VI) and Alizarin Red S dye by Fe_3_O_4_@Talc nanocomposite have been scarce. In recent years, several studies have investigated the fabrication of adsorbent Fe_3_O_4_@ Talc nanomaterials for use in wastewater treatment [34].

In the literature, previous works investigating the adsorption of Cr(VI) and Alizarin Red S dye using Fe_3_O_4_@Talc nanocomposite from concentrated acidic media with considerable adsorption capacities are limited. On the other hand, due to the unique properties of magnetite talc adsorbents, they have great efficiency in adsorbing both organic and inorganic pollutants and can be prepared using environmentally friendly processes. In addition, due to the magnetic properties of Fe_3_O_4_@Talc nanocomposite, it can be easily separated from adsorption media [34]. Therefore, the use of magnetite nanocomposite adsorbent material is an excellent way to resolve separation problems [31]. Moreover, these magnetic properties enhance the use of Fe_3_O_4_@Talc nanocomposites in adsorption processes involving the combination of electrostatic attraction and ligand exchange [31].

Therefore, in this study an Fe_3_O_4_@Talc nanocomposite adsorbent was fabricated from synthetic Talc to adsorb Cr(VI) ions and ARS dye from acidic aqueous media. The prepared nanocomposite was characterized by SEM, FTIR, XRD, and TGA. Different parameters affecting the adsorption processes were investigated. Moreover, the isothermics and kinetics of the adsorption of both Cr(VI) ions and (ARS) dye from aqueous media by Fe_3_O_4_@Talc nanocomposite were investigated, and the regeneration and reuse of saturated Fe_3_O_4_@Talc nanocomposite was studied.

## 2. Experimental

### 2.1. Materials

All chemicals and materials were of analytical grade and used as received without further purification. Ferric chloride hexahydrate (FeCl_3_.6H_2_O ≥ 98%), ferrous sulfate heptahydrate (FeSO_4_.7H_2_O ≥ 99%), and ammonia solution 25% were obtained from Sigma Aldrich, Missouri, USA, and potassium dichromate (K_2_Cr_2_O_7_) was purchased from Merck, Darmstadt, Germany.

The pH values were adjusted by the addition of NaOH or HCl, which was obtained from Fluka Gillingham, England. Alizarine Red-S was purchased from Sigma Aldrich. Talc powder (magnesium hydroxysilicate) of pharmaceutical grade was purchased from a local market. All experiments were carried out at ambient temperature unless otherwise stated.

### 2.2. Preparation of Fe_3_O_4_@Talc Nanocomposite

Briefly, 3.50 g of talc was suspended in 400.0 mL water, then 6.40 g of FeCl_3_ and 5.56 g of FeSO_4_.7H_2_O (at a molar ratio of Fe^3+^:Fe^2+^ = 2:1) was added. The mixture was stirred for 1.0 h for impregnation on the surface of talc powder, then heated to 80 °C. Ammonia solution 25% was added drop wise under an N_2_ atmosphere until pH 9.5 was reached. The black precipitate was collected by filtration and washed with deionized water, then again with ethanol, then dried at 60 °C overnight, and finally stored in a sealed bottle. All experiments were conducted using (0.05 g/10 mL) Fe_3_O_4_@Talc nanocomposite at optimum conditions of (Cr(VI)) = 10 mg/L, contact time = 30 min, pH = 3, T °C = 20 °C for Cr(VI) and ARS dye = 50 mg/L, contact time = 60 min, pH = 2, T °C = 20 °C for ARS dye, unless otherwise stated.

### 2.3. Characterization of Fe_3_O_4_@Talc Nanocomposite

The physicochemical characteristics of the magnetite talc (Fe_3_O_4_@Talc) nanocomposite were investigated. The surface structure of the investigated adsorbent was conducted via scanning electron microscopy (SEM) on a Thermo Scientific Quattro ESEM (Thermo Fischer, Waltham, MA, USA). Thermal stability was investigated using a Shimadzu TGA 51- thermal analyzer at a heating rate of 10 °C/min (Thermofisher Scientific, Waltham, MA, USA).

The X-ray diffraction (XRD) pattern of the investigated composite was investigated using a Shimadzu X-ray Diffractometer, XRD-7000 “Shimadzu, Kyoto, Japan” with a nickel filter and a CuK_α_-X-ray tube (λ = 1.5418 Å) at a diffraction angle (2θ) range of 10–80° and scan speed of 0.2°/min. The Attenuated Total Reflection-Fourier Transform Infrared (ATR-FTIR) spectrum was recorded using an IRTracer-100 from Shimadzu, Kyoto, Japan, in order to identify the functional groups. The sample was examined in the range from 4000 to 400 cm^−1^ at a resolution of 4.0 cm^−1^.

### 2.4. Adsorption Studies

The concentration of Cr(VI) was measured spectrophotometrically via the 1,5- Diphenyl carbazide method. The absorbance was measured at 543 nm against blank reagent, while the concentration of Alizarine Red-S (ARS)-dye was measured at 505 nm.

Removal efficiency (%R) is defined as

(1) $$\%R=\frac{\left(C_{o}-C_{t}\right)}{C_{o}}\times 100$$

where, C_o_ and C_t_ are the initial concentration and the concentration at time t, respectively.

### 2.5. Regeneration and Reuse Studies of Adsorbent

In order to study and understand their respective regeneration cycles, the desorption process was studied separately for Cr(VI) and (ARS)-dye. The saturated Fe_3_O_4_@Talc nanocomposite was rinsed with 10.0 mL of 0.001 M of NaOH, HCl, or HNO_3_. In the reusability study, the saturated Fe_3_O_4_@Talc nanocomposite was washed with ten mL (0.001) NaOH solution four times for Cr(VI) and six times for (ARS)-dye, then washed with deionized water to remove the excess alkaline solution, and finally dried for reuse.

## 3. Results and Discussion

### 3.1. Characterization of Fe_3_O_4_@Talc Nanocomposite

#### 3.1.1. SEM

**Figure 1:**
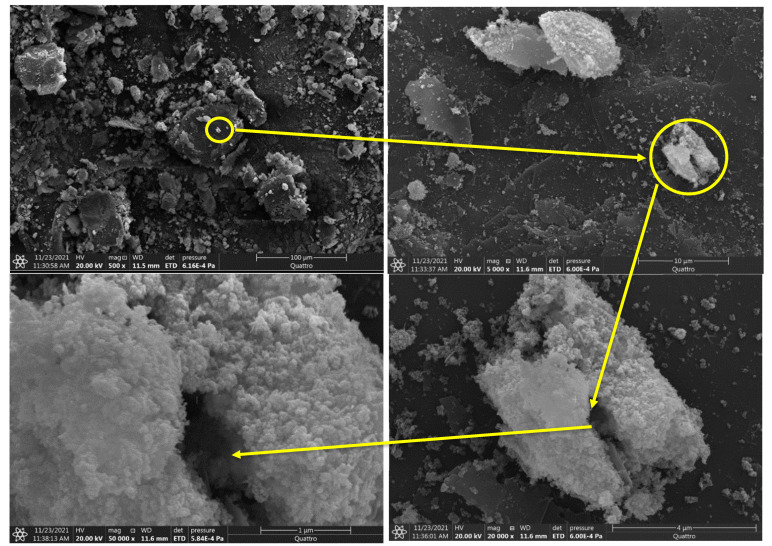
SEM images of the prepared magnetite-Talc nanocomposite at different magnifications values (500, 5000, 20,000, and 50,000×).

The morphology of Fe_3_O_4_@Talc nanocomposite was analyzed, and is represented in Figure 1. Figure 1 illustrates that the available surface of the talc layers was covered by spherical magnetic nanoparticles [30,34]. The prepared nanocomposite appears as flatter blocks and thick cleaved platelets. High magnification values show that on the external surface of the nanocomposite the small magnetic nanoparticles are aggregated together on the accessible surfaces of the talc to create large particles with a shiny appearance.

#### 3.1.2. FTIR Analysis

**Figure 2:**
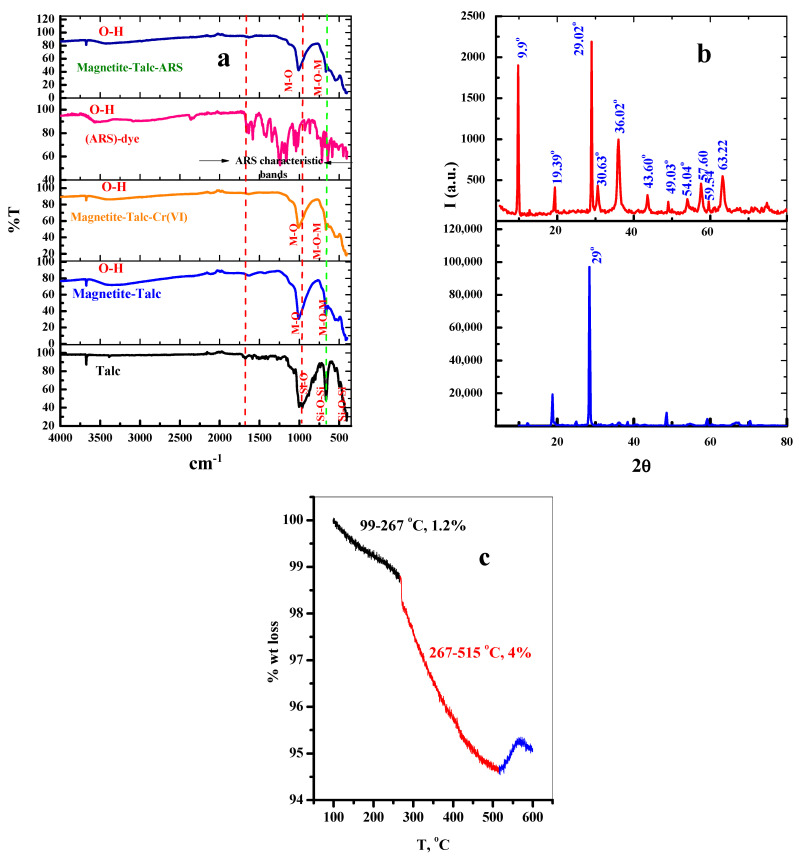
(**a**) FTIR spectra of the prepared magnetite-Talc nanocomposite and its complexes with Cr(VI) and Alizarin red S (ARS)-dye; (**b**) XRD pattern of prepared magnetite-Talc nanocomposite; and (**c**) TGA images of prepared magnetite-Talc nanocomposite.

Figure 2a represents the FT-IR spectra for the magnetite talc nanocomposite and its complexes with Cr(VI) and Alizarin red S (ARS)-dye, with the wavenumber varying from 4000 to 400 cm^−1^.

For Talc, the characteristic peaks at 3674, 1011, 667, 523, and 461 cm^−1^ are due to stretching vibrations of the structural hydroxyl moiety Mg-OH, Si-O, Si-O-Mg, Mg-O, and the Si-O-Si bands of talc [27,30,32,35]. The peak appearing at 1624 cm^−1^ is attributed to the stretching vibrations of the strong H-bond-OH inside talc or adsorbed H_2_O [27], and the peaks at 1070 and 962 cm^−1^ are ascribed to the out- and/or in-plane stretching of Si-O peaks [27]. The vibrational bands appearing in the range of 400–900 cm^−1^ are associated with the asymmetric Si-O-Si, Si-O-Mg, SiO flexural vibrations [34,35,36].

After the modification of talc with Fe_3_O_4_, a small and sharp band at 3676 cm^−1^ is attributed to the inner-surface O-H group stretching mode [36]. Stretching vibrational bands of the siloxane group (Si-O-Si) appeared at 1070 and 461 cm^−1^, and can be assigned to the presence of a silica layer around the core of the Fe_3_O_4_ nanoparticles [36]. The observed peak at 550 cm^−1^ is attributed to the stretch vibration band of Fe-O in the Fe_3_O_4_-NPs [37]. On the other hand, most of the characteristic peaks of talc are reduced in the spectra of the Fe_3_O_4_@Talc nanocomposite due to the effects of ordering and bonding on the layer structures; the other interlayer bonds remain unchanged in the talc [34,35,36]. This confirms the formation of nano-Fe_3_O_4_ on the exterior surfaces of the talc sample [38,39].

After the adsorption processes of Cr(VI) ions and (ARS)-dye it is obvious from Figure 2a that the most characteristic adsorption peaks of Fe_3_O_4_@Talc nanocomposite, such as the Mg-OH, Si-O, Si-O-Mg, Mg-O, Si-O-Si, and Fe_3_O_4_-NPs bands, were reduced, as these functional groups are partially masked by the species of Cr(VI) and (ARS)-dye following adsorption [38,39].

#### 3.1.3. XRD Analysis

The XRD analyses for Talc and the prepared Fe_3_O_4_@Talc nanocomposite are illustrated in Figure 2b. According to the PDF 19-0770 powder diffraction file [40], strong characteristic diffraction peaks at 2θ = 29° show the presence of quartz, while other peaks indicate the presence of talc (Mg_3_Si_4_O_10_(OH)_2_) [27,40].

For the Fe_3_O_4_@Talc nanocomposite sample, the positions and intensities of all characteristic diffraction peaks of the Fe_3_O_4_@Talc nanocomposite were obtained. Due to the cubic spinel structure of the magnetic nanoparticles, various diffraction peaks were observed at 2θ = 9.9, 19.39, 29.02, 30.63, 36.02, 37.64, 49.03, 54.05, 55.43, 57.6, 59.55, 61.01, 63.23, 70.82, 72.02, 73.37, and 74.72 [30,34]. All of these peak positions at 2θ = 9.9 (001), 19.39 (111), 29.02 (003), 30.63 (003), 36.02 (311), 57.6 (511), 59.55, and 61.01 (440) are in agreement with the standard X-ray data on Fe_3_O_4_@Talc nanocomposites [34]. The decrease in density of the characteristic peaks of Talc after the formation of the Fe_3_O_4_@Talc nanocomposite can be attributed to the formation of magnetite nanoparticles on the external surfaces of the Talc layer [34].

These results are in good agreement with those previously obtained [30,34] and observed in Figure 1, where in the prepared nanocomposite the Fe_3_O_4_ nanoparticles were at most immobilized as an external layer on the outer surface of the talc [30].

#### 3.1.4. Thermogravimetric Analysis (TGA)

TGA analysis of Fe_3_O_4_@Talc nanocomposite is presented in Figure 2c, showing the quality loss rate (% weight loss) of the prepared nanocomposites at temperatures up to 600 °C, which was investigated to study the thermal stability of the prepared nanocomposite. About 5.2% weight loss is observed with increasing temperatures to about 600 °C. The fabricated Fe_3_O_4_@Talc nanocomposite illustrates two decomposition steps, which can be easily characterized. In the first, the weight loss (1.2%) in the temperature range (99.0–267 °C) can be attributed the vaporization of water molecules and volatilization of other impurities [39]. In the second, the weight loss occurring at the temperature range of 267–515 °C was 4.0%, which this may due to the loss of H-bonds in water or (-OH)-groups in the Fe_3_O_4_@Talc nanocomposite structure [36]. When further increasing the temperature to 600 °C, a slight increment could be observed. This slight increment peak centered at 571 °C can be attributed to formation of haematite and the reactions between metal oxides (such as haematite) and H_2_O molecules resulting from the dehydroxylation of talc powder [40]. The low weight loss observed in this temperature range (about 5.2%) indicates the thermal stability of the prepared Fe_3_O_4_@Talc nanocomposite.

### 3.2. Adsorption Processes

#### 3.2.1. Effect of Initial Concentrations

**Figure 3:**
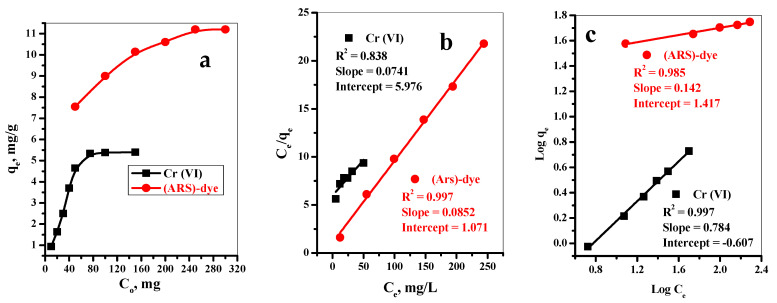
(**a**) Effect of initial concentrations of Cr(VI) and (ARS)-dye, (**b**) Freundlich, and (**c**) Freundlich isotherm models for adsorption of Cr(VI) and (ARS)-dye on adsorption capacity onto 0.05 g/10 mL Fe_3_O_4_@Talc nanocomposite. For Cr(VI), contact time = 30 min, pH = 3, T = 20 °C. For ARS-dye, contact time = 60 min, pH = 2, T = 20 °C.

The influence of the initial concentrations of both Cr(VI) and (ARS)-dye on their adsorption capacities by the Fe_3_O_4_@Talc nanocomposite were investigated in the ranges of 10–50 mg/L and 10–250 mg/L for Cr(VI) and (ARS)-dye, respectively, as shown in Figure 3a. We observed that the adsorption capacities of both Cr(VI) and (ARS)-dye were dramatically increased with further increases in their initial concentrations until reaching steady state. This was due to the number of contaminant species being very low at low contaminant concentrations compared with the number of binding sites. This can be explained by the fact that the initial Cr(VI) ions and (ARS)-dye concentrations provided a driving force to overcome the mass transfer resistance between the Fe_3_O_4_@Talc nanocomposite solid phase and the (Cr(VI) and (ARS)-dye aqueous phases [41]. On the other hand, with further increases in the concentration of contaminants, the number of binding sites becomes insufficient for the capture of all contaminant species.

The adsorption isotherms describe the relationship among the concentrations of Cr(VI) ions and (ARS)-dye and deposition degree on the surface of the Fe_3_O_4_@Talc nanocomposite at constant temperature. The most widely used isotherm models are Langmuir (which supposes that the adsorbent has the same active sites and that Cr(VI) ions and (ARS)-dye form a uniform layer on the adsorbent surface) and Freundlich (which supposes that active adsorbent sites with different energies and multiple layers of Cr(VI) ions and (ARS)-dye are involved); see Equations (S1)–(S3) in the Supplementary Materials. The linearized form plots of the Langmuir (C_e_ vs. C_e_/q_e_) and Freundlich (log C_e_ vs. log q_e_) models are shown in Figure 3b,c, respectively, where, q_e_ is the amounts of Cr(VI) ions and (ARS)-dye per unit mass of Fe_3_O_4_@Talc nanocomposite (mg·g^−1^) and C_e_ is the concentration of Cr(VI) ions or (ARS)-dye at equilibrium (mg·L^−1^). The relationship coefficient and factors related to the two models were calculated and are listed in Table 1. For the removal of Cr(VI) by Fe_3_O_4_@Talc nanocomposite, the (R^2^ = 0.997) related to the Freundlich isotherm is much higher than that related to the Langmuir isotherm (R^2^ = 0.838), which shows that the adsorption of Cr(VI) is better fitted by the Freundlich model than by the Langmuir model.

These data illustrate the superiority of the Freundlich model and show that the adsorption process of Cr(VI) using Fe_3_O_4_@Talc nanocomposite occurred on the non-uniform surfaces of the nanocomposite in multilayered adsorption forms [42]. On the other hand, the adsorption of (ARS)-dye by the Fe_3_O_4_@Talc nanocomposite was excellently described by the Langmuir model (R^2^ = 0.997). This indicates that homogeneous adsorption of (ARS)-dye by the Fe_3_O_4_@Talc nanocomposite occurred, as well as the homogenous nature of the nanocomposite surfaces [41]. The calculated values of R_L_ were equal to 0.89 and 0.2 for Cr(VI) and (ARS)-dye, respectively; i.e., 0 ˂ R_L_ ˂ 1, meaning that the investigated adsorption processes of Cr(VI) and (ARS)-dye by the Fe_3_O_4_@Talc nanocomposite were favorable.

#### 3.2.2. Adsorption Kinetics

The effect of contact time on the adsorption capacities of Cr(VI) and (ARS)-dye by 0.05 g Fe_3_O_4_@Talc nanocomposite were studied for a period of 2.0–40.0 min at 25–55 °C with initial concentrations of 10.0 and 50 mg·L^−1^ for Cr(VI) and (ARS)-dye, respectively. Figure S1 (Supplementary Materials) represents the data obtained to evaluate the kinetic behaviors of the adsorption processes of Cr(VI) and (ARS)-dye by Fe_3_O_4_@Talc nanocomposite using the pseudo-first-order, pseudo-second-order, intra-particle diffusion, and Elovich models, as in Equations (2)–(5), respectively [41,43,44]. The adsorption type (chemical or physical adsorption) can be determined by the pseudo-first or pseudo-second order models. On the other hand, the transport and adsorption of Cr(VI) and (ARS)-dye onto the Fe_3_O_4_@Talc nanocomposite can be explained by the intra-particle diffusion and Elovich models [44].

For the pseudo-first-order model,

(2) $$\log(q_{e}-q_{t})=\log q_{e}-(\frac{k_{1}}{2.303})t$$

For the pseudo-second-order model,

(3) $$\left(\frac{t}{q_{t}}\right)=\left(\frac{1}{k_{2}q_{e}^{2}}\right)+\left(\frac{1}{q_{e}}\right)\;t$$

For the Elovich model,

q_t_ = 1/b ln(ab) + 1/b lnt (4)

For the intra-particle diffusion model,

q_t_ = k_i_t^1/2^ + C (5)

where q_e_ and q_t_ (mg/g) are the amounts of Cr(VI) ions and (ARS)-dye sorbed by Fe_3_O_4_@Talc nanocomposite at equilibrium and at time (t), respectively and k_1_ (min^−1^) and k_2_ (g·mg^−1^·min^−1^) are the rate constants for pseudo-first and pseudo-second-order, respectively.

Here, k_i_ (mg·g^−1^·min^−0.5^) refers to the intra-particle diffusion rate constant and C is the intercept that is proportionate to the boundary layer thickness. The constants “a” (mg/g·min) and (b) are the initial adsorption rate and constant characteristic of the adsorption processes, respectively, while q_t_ is the amount of Cr(VI) and (ARS)-dye sorbed by the Fe_3_O_4_@Talc nanocomposite at time (lnt, min) [44].

For the pseudo-first order model, by plotting log (q_e_ − q_t_) versus (t) the values of k_1_ and q_e_ can be calculated, as represented in Figure S1a and Table 2. The pseudo-second order constants are determined by plotting t/q_t_ against t, as shown in Figure S1b and Table 1. In addition, for the Elovich model the relationships between q (mg/g) against lnt are shown in Figure S1c, while for the intra-particle diffusion model the relationships between q (mg/g) and t^1/2^ is illustrated in Figure S1d. The values of the constants were estimated for both the Elovich and intra-particle diffusion models, as shown in Table 2.

#### 3.2.3. Effect of Adsorbent Dosage

**Figure 4:**
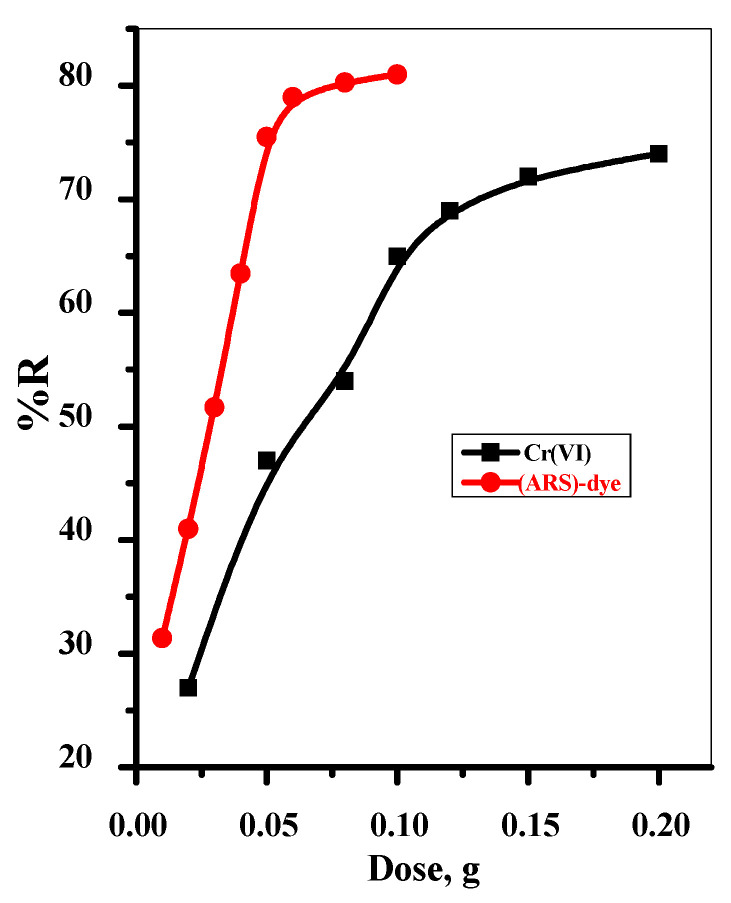
The effect of adsorbent dosage on the removal of Cr(VI) and Alizarin red S (ARS)-dye with 0.05 g/10 mL Fe_3_O_4_@Talc nanocomposite.

The Fe_3_O_4_@Talc nanocomposite dose is a highly influential factor with respect to adsorption performance. The effect of adsorbent dose on the adsorption percentages of both Cr(VI) and (ARS)-dye were investigated with initial concentrations of 10.0 and 50 mg/L for Cr(VI) and (ARS)-dye and contact times of 30 and 60 min, respectively, as illustrated in Figure 4. It can be clearly seen that the removal efficiencies of both pollutants were enhanced with further increases in the adsorbent dose. This can be attributed to increases in the Fe_3_O_4_@Talc nanocomposite dose, providing more active sites to interact with more Cr(VI) and (ARS)-dye species, resulting in improved adsorption efficiency.

#### 3.2.4. Effect of Initial pH

**Figure 5:**
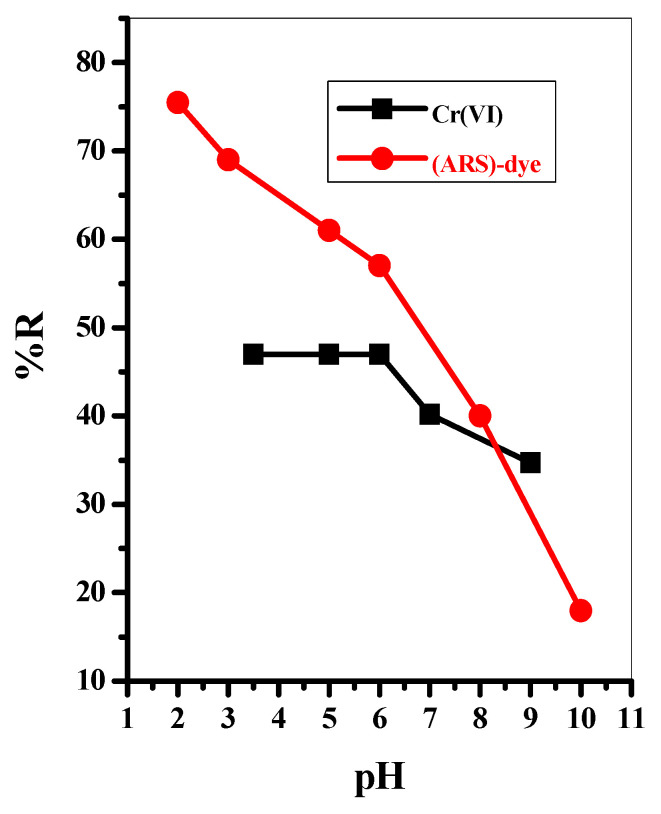
The effect of pH on the removal of Cr(VI) and Alizarin red S (ARS)-dye with 0.05 g/10 mL Fe_3_O_4_@Talc nanocomposite.

The pH factor is a key parameter influencing the adsorption process; it influences the surface of the Fe_3_O_4_@Talc nanocomposite and the chemistry of the aqueous medium. Therefore, the impact of pH on the adsorption percentages (%R) of both Cr(VI) and (ARS)-dye were investigated at various pH values (2.0–10 for (ARS)-dye and 3.0–9.0 for Cr(VI)) with 0.05 g Fe_3_O_4_@Talc nanocomposite, as represented in Figure 5. The %R values of both Cr(VI) and (ARS)-dye were higher, gradually decreasing with increasing pH of the solution, with the pH influencing the surface of the Fe_3_O_4_@Talc nanocomposite as well as Cr(VI) and (ARS)-dye dissociation.

The face of layers of the Fe_3_O_4_@Talc nanocomposite are composed of fully exposed O-atoms, which have a very low electrical charge and are nonpolar and hydrophobic [45]. Therefore, at low pH values the surface of the Fe_3_O_4_@Talc nanocomposite are positively charged due to protonation of its binding sites, which interact favorably with the negatively charged predominant anionic species of Cr(VI) and the sulfonate groups of (ARS)-dye [34,43], causing an increase in adsorption percentage. As the pH value increases, the number of H^+^ ions decrease and the negativity of the binding sites increases, which is repulsed by the Cr(VI) and (ARS)-dye species, leading to a decrease in removal efficiency.

At lower pH values, the most predominant species of Cr(VI) ions is HCrO_4_^−^, while in alkaline and neutral conditions CrO_4_^2−^ is the dominant species. HCrO_4_^−^ species are more electrostatically attracted and more favorably adsorb on the positive surface of Fe_3_O_4_@Talc nanocomposite than CrO_4_^2−^ species [22]. This can be attributed to the higher oxidizing capacity and lower adsorption free energy of HCrO_4_^−^ species [22]. On the other hand, at lower pH values (ARS)-dye can be removed at the surfaces of Fe_3_O_4_@Talc nanocomposite through the *β*-phenolic oxygen, while with increasing pH value the negative -OH groups increase and compete with negatively charged (ARS)-dye anions for adsorption on the active sites, leading to a reduction in the adsorption percentage of (ARS)-dye [43,46].

#### 3.2.5. Effects of Temperature

The effect of temperature on the adsorption of Cr(VI) and (ARS)-dye by the Fe_3_O_4_@Talc nanocomposite in the range of 20–50 °C was carefully investigated while keeping all other experimental conditions constant. When increasing the temperature from 293 to 325 K, the adsorption percentage (%R) of Cr(VI) was decreased to 38%, while the %R of (ARS)-dye increased to about 80%. This can be attributed to the fact that with increasing temperature the mobility of the Cr(VI) ions increases, causing desorption, and the solution viscosity increases leading to an enhanced diffusion rate of (ARS)-dye at the exterior boundary layers and within the pores of the Fe_3_O_4_@Talc nanocomposite [47].

#### 3.2.6. Thermodynamics

**Figure 6:**
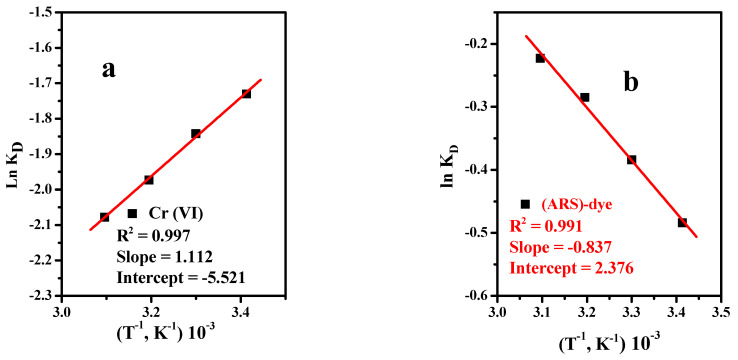
Relationship between lnK_d_ and 1/*T* for the (**a**) Cr(VI) and (**b**) Alizarin red S (ARS)-dye with 0.05 g/10 mL Fe_3_O_4_@Talc nanocomposite.

The values of the thermodynamic factors, involving the Gibbs free energy (Δ*G°*), enthalpy (Δ*H°*), and entropy (Δ*S°*) were determined using Equations (6) and (7), and the value of the logarithmic plot of the distribution coefficient *K_d_* vs. 1/*T* is presented in Figure 6. The calculated values of Δ*G°*, Δ*H°*, and Δ*S°* are summarized in Table 3.

(6) $$Ln\;K_{d\;}=\frac{\Delta S}{R}-\frac{\Delta H}{RT}$$

(7) $$\Delta G=-RT\;Ln\;K_{d}$$

where (Δ*G°*) describes the variation value of the Gibbs free energy during the process (kJ·mol^−1^), (Δ*H°*) illustrates the alternate values of enthalpy during the process (kJ·mol^−1^), (Δ*S°*) represents the entropy change value of the process (J·mol^−1^·K^−1^), R represents the gas constant (8.314 J·mol^−1^·K^−1^), and T represents the absolute temperature (K).

As observed in Table 3, the value of enthalpy (Δ*H*) was negative in case of Cr(VI), revealing the exothermic adsorption reactions, while it was positive in the case of (ARS)-dye, indicating endothermic adsorption reactions. Moreover, the entropy (Δ*S°*) possessed a negative value for Cr(VI), as there was decrease in the degree of randomness at the solid/solution interface. On the other hand, it had a positive value in the case of (ARS)-dye (0.0198 kJ·mol^−1^·K), indicating randomness in the adsorption system. The negative values of (Δ*G°*) emphasize that the investigated adsorption processes of Cr(VI) and (ARS)-dye by the Fe_3_O_4_@Talc nanocomposite have a spontaneously nature at various temperatures [1].

### 3.3. Regenaration and Reuse

The adsorption processes, regeneration, and reuse of the applied adsorbent are very important parameters in determining its economic viability and practical applications [43]. Therefore, regeneration and reuse of Fe_3_O_4_@Talc nanocomposite were studied using different alkaline and acidic solutions. The results obtained showed that more than 94% of Cr(VI) and (ARS)-dye was desorbed with 0.001 M NaOH solution. This can be attributed to the anionic characteristics of Cr(VI) ions and (ARS)-dye and the positive active sites of the nanocomposite, where replacement reactions with negative OH^−^ ions of the eco-friendly desorption solvent NaOH occurred [43].

The reusability cycles were investigated up to four and six times for Cr(VI) and (ARS)-dye, respectively. Then, the adsorption capacities of the regenerated Fe_3_O_4_@Talc nanocomposite for each adsorbate were estimated. The data obtained illustrated that the adsorption efficiencies of Fe_3_O_4_@Talc nanocomposite for Cr(VI) and (ARS)-dye remained higher than 89% and 92% after four and six cycles of regeneration, respectively. This means that the prepared Fe_3_O_4_@Talc nanocomposite is a stable adsorbent with the property of reusability.

### 3.4. Comparison of (Fe_3_O_4_@Talc) Nanocomposite Adsorption Efficiency with Other Adsorbents

A comparison of the adsorption of Cr(VI) and Alizarin Red S dye onto the prepared magnetite talc (Fe3O4@Talc) nanocomposite and other adsorbent materials published recently is shown in Table 4 [24,34,48,49,50,51,52,53,54,55]. The data obtained clearly show the advantages of the prepared Fe_3_O_4_@Talc nanocomposite over many other adsorbents for the adsorption of Cr(VI) and Alizarin Red S dye, especially under acidic conditions, as well as its lower contact time and considerable maximum adsorption capacity (Q_max_, mg/g).

## 4. Conclusions

This work represents the successful fabrication of an Fe_3_O_4_@Talc nanocomposite from Talc powder as an effective adsorbent for Cr(VI) and (ARS)-dye. The prepared composite was characterized by SEM, TGA, FTIR, and XRD in order to study its physical and chemical properties. Under optimal conditions, the maximum adsorption capacity of Fe_3_O_4_@Talc toward Cr(VI) and (ARS)-dye was 13.5 and 11.76 mg·g^−1^, respectively, and followed the pseudo-second order model. The thermodynamic parameters showed the investigated adsorption processes of Cr(VI) and (ARS)-dye sorbed by Fe_3_O_4_@Talc nanocomposite to be an exothermic adsorption reaction for Cr(VI) and an endothermic reaction for (ARS)-dye. A regeneration study of the Fe_3_O_4_@Talc nanocomposite showed that it has considerable removal efficiency even in the sixth regeneration cycle. Therefore, the prepared Fe_3_O_4_@Talc nanocomposite can be regarded as a promising material that can be used to treat wastewater from various pollutants.

## Figures and Tables

**Table 1:** Calculated equilibrium constants for the adsorption of Cr(VI) ions and (ARS)-dye onto 0.05 g/10 mL Fe_3_O_4_@Talc nanocomposite.

Adsorbate	Langmuir Isotherm Model				Freundlich Isotherm Model		
	Q_max_ (mg/g)	b (L/mg)	R_L_	R^2^	1/n	K_f_ (mg/g)	R^2^
Cr(VI) (ARS)-dye	13.50 11.76	0.0124 0.080	0.89 0.2	0.838 0.997	0.784 0.142	0.247 13.860	0.997 0.985

**Table 2:** The calculated parameters of the pseudo-first order, pseudo-second order, intra-particle diffusion, and Elovich kinetic models of adsorption of Cr(VI) and (ARS)-dye ions onto 0.05 g/10 mL Fe_3_O_4_@Talc nanocomposite.

Adsorbate	T, °C	q_e exp_ (mg/g)	First-Order Kinetic Parameter			Second-Order Kinetic Parameter			Intra-Particle Diffusion			Elovich		
			K_1_ (min^−1^)	q_e cal_ (mg/g)	R^2^	K_2_ (g·mg^−1^·min^−1^)	q_e·cal_ (mg/g)	R^2^	K_i_	C	R^2^	a	b	R^2^
Cr(VI)	20	0.940	−0.085	0.977	0.969	0.05	1.314	0.902	0.194	−0.1136	0.96	0.209	3.992	0.942
	30	0.884	−0.085	0.948	0.947	0.046	1.28	0.837	0.185	−0.1237	0.990	0.188	4.181	0.946
	40	0.820	−0.081	0.867	0.952	0.051	1.163	0.805	0.171	−0.1212	0.990	0.171	4.608	0.944
	50	0.770	−0.071	0.800	0.933	0.058	1.013	0.720	0.159	−0.130	0.976	01.52	5.276	0.945
(ARS)-dye	20	7.55	−0.047	9.42	0.932	1.99 × 10^−4^	31.25	0.818	1.334	−2.334	0.992	0.591	0.399	0.944
	30	7.73	−0.044	8.79	0.941	8.94 × 10^−4^	16.67	0.924	1.225	−1.74	0.992	0.674	0.423	0.947
	40	7.90	−0.041	8.17	0.953	2.39 × 10^−3^	11.78	0.946	1.11	−1.182	0.990	0.784	0.444	0.956
	50	8.0	−0.0345	7.13	0.963	4.42 × 10^−3^	9.80	0.931	1.101	−0.571	0.978	0.931	0.480	0.945

**Table 3:** Thermodynamic parameters for adsorption of Cr(VI) and (ARS)-dye ions onto 0.05 g/10 mL Fe_3_O_4_@Talc nanocomposite.

T (K)	ΔG° (kJ·mol^−1^)		ΔH° (kJ·mol^−1^)		ΔS° (J·mol^−1^·K^−1^)	
	Cr(VI)	(ARS)-Dye	Cr(VI)	(ARS)-Dye	Cr(VI)	(ARS)-Dye
293	−4.21	−1.18	−9.24	6.96	−46.0	19.8
303	−4.64	−0.97				
313	−5.13	−0.74				
323	−5.58	−0.60				

**Table 4:** Comparison of adsorption capacities for Cr(VI) using various adsorbent materials.

Adsorbent	Cr(VI)				(ARS)-Dye				Ref.
	Q_max_, mg/g	pH	°C	Time (min)	Q_max_, mg/g	pH	°C	Time (min)	
acetic acid modified clay (AMC)	10.42	6		90					[24]
hydrochloric acid modified clay (HMC)	18.15	7		50					
Fe_3_O_4_/Talc nanocomposite	7.17	5	20	60					[34]
	7.47		30						
oxalic acid reduction-modified fly ash (MFA)	12.34			120					[48]
Magnetic Goethite	4.32	4	30	240					[49]
activated carbon supported iron catalysts (Fe5-AWS)	10.11	2		60					[50]
Fe3O4–PEI800–MMT	8.8	6	25	120					[51]
Fe3O4–PEI25000–MMT	7.7	6	25	120					[51]
GO-MA_30:1_ composite	13.6			10					[52]
Fe_3_O_4_/Talc nanocomposite	13.5	2	20	30					This Work
mesoporous carbon					2923	6		80	[53]
polyquinone/graphene					3290			120	
Spirulina platensis					17.15	6.47		46.29	[54]
Maghemite Fe_2_O_3_					23.2	11		60	[55]
					11.9	8		60	
Fe_3_O_4_/Talc nanocomposite					11.7	3	20	60	This Work

## Data Availability

Data is contained within the article.

## Supplementary Materials

The following supporting information can be downloaded at: https://www.mdpi.com/article/10.3390/ma15093401/s1, Figure S1: The kinetic models of adsorption for adsorption of both Cr(VI) and (ARS)-dye onto 0.05g/10mL Fe_3_O_4_@Talc nanocomposite, (**a**) Pseudo-first order, (**b**) and Pseudo-second order, (**c**) Elovich model, and (**d**) intraparticle diffusion.
