# Supplementary material for: Preparation and Characterization of Magnetite Talc (Fe3O4@Talc) Nanocomposite as an Effective Adsorbent for Cr(VI) and Alizarin Red S Dye
Source: Materials (Basel). 2022 May 9;15(9):3401. doi: 10.3390/ma15093401 (PMC9105966; doi:10.3390/ma15093401)
Supplement: Supplementary file 1 [file materials-15-03401-s001.zip › materials-1699329-supplementary.pdf]

*materials* MDPI

*Supplementary Materials*

# Preparation and Characterization of Magnetite Talc ($Fe_3O_4$@Talc) Nanocomposite as an Effective Adsorbent for Cr(VI) and Alizarin Red S Dye

**AbdElAziz A. Nayl [1,*], Ahmed I. Abd-Elhamid [2], Ismail M. Ahmed [1] and Stefan Bräse [3,4,*]**

1 Department of Chemistry, College of Science, Jouf University, Sakaka 72341 , Al Jouf, Saudi Arabia; aanayel@ju.edu.sa or aanayl@yahoo.com (A.A.N.); ismadwy@yahoo.ca (I.M.A.)
2 Nanotechnology and Composite Materials Research Department, Advanced Technology and New Materials Research Institute (ATNMRI), City of Scientific Research and Technological Applications (SRTA-City), New Borg Al-Arab, Alexandria 21934, Egypt ; ahm_ch_ibr@yahoo.com
3 Institute of Organic Chemistry (IOC), Karlsruhe Institute of Technology (KIT), Fritz-Haber-Weg 6, 76133 Karlsruhe, Germany
4 Institute of Biological and Chemical Systems—Functional Molecular Systems (IBCS-FMS), Director Hermann-von-Helmholtz-Platz 1, D-76344 Eggenstein-Leopoldshafen, Germany
* Correspondence: aanayel@ju.edu.sa or aanayl@yahoo.com (A.A.N.); stefan.braese@kit.edu (S.B.)

***In Langmuir isotherm model,*** the linear form is represented by the following equation:

$$\frac{1}{q_e} = \frac{1}{Q_{\max}} + \frac{1}{bQ_{\max}}\left(\frac{1}{C_e}\right) \quad \text{(S1)}$$

where, $q_e$ is the amounts of Cr(VI) ions and (ARS)-dye per unit mass of $Fe_3O_4$@Talc nanocomposite ($mgg^{-1}$), $C_e$ is the Cr(VI) ions and (ARS)-dye concentrations at equilibrium ($mgL^{-1}$), and $Q_{max}$ and b are Langmuir constants related to the sorption capacities and binding energies between the sorbent with the sorbate, respectively.

One of the essential characteristics of Langmuir isotherm is a dimensionless constant that is called separation factor or equilibrium parameter $R_L$ and can be estimated by the following equation;

$$R_L = \frac{1}{1 + bC_o} \quad \text{(S2)}$$

The value of separation factor $R_L$ indicates either the adsorption isotherm to be unfavorable ($R_L > 1$), favorable ($0 < R_L > 1$), irreversible (RL=0) or linear (RL=1).

***The Freundlich model*** is an empirical equation used to describe the sorption occurred on heterogeneous surfaces. The linear equation is commonly demonstrated as:

$$\log q_e = \log K_f + (1/n)\log Ce \quad \text{(S3)}$$

where, $q_e$ ($mgg^{-1}$) and $C_e$ ($mgL^{-1}$) are the amounts of Adsorbate per unit mass of $Fe_3O_4$@Talc nanocomposite and Adsorbate concentrations at equilibrium, respectively. $K_f$ and 1/n are the Freundlich model constants that indicate to the sorption capacities and the sorption intensities, respectively.

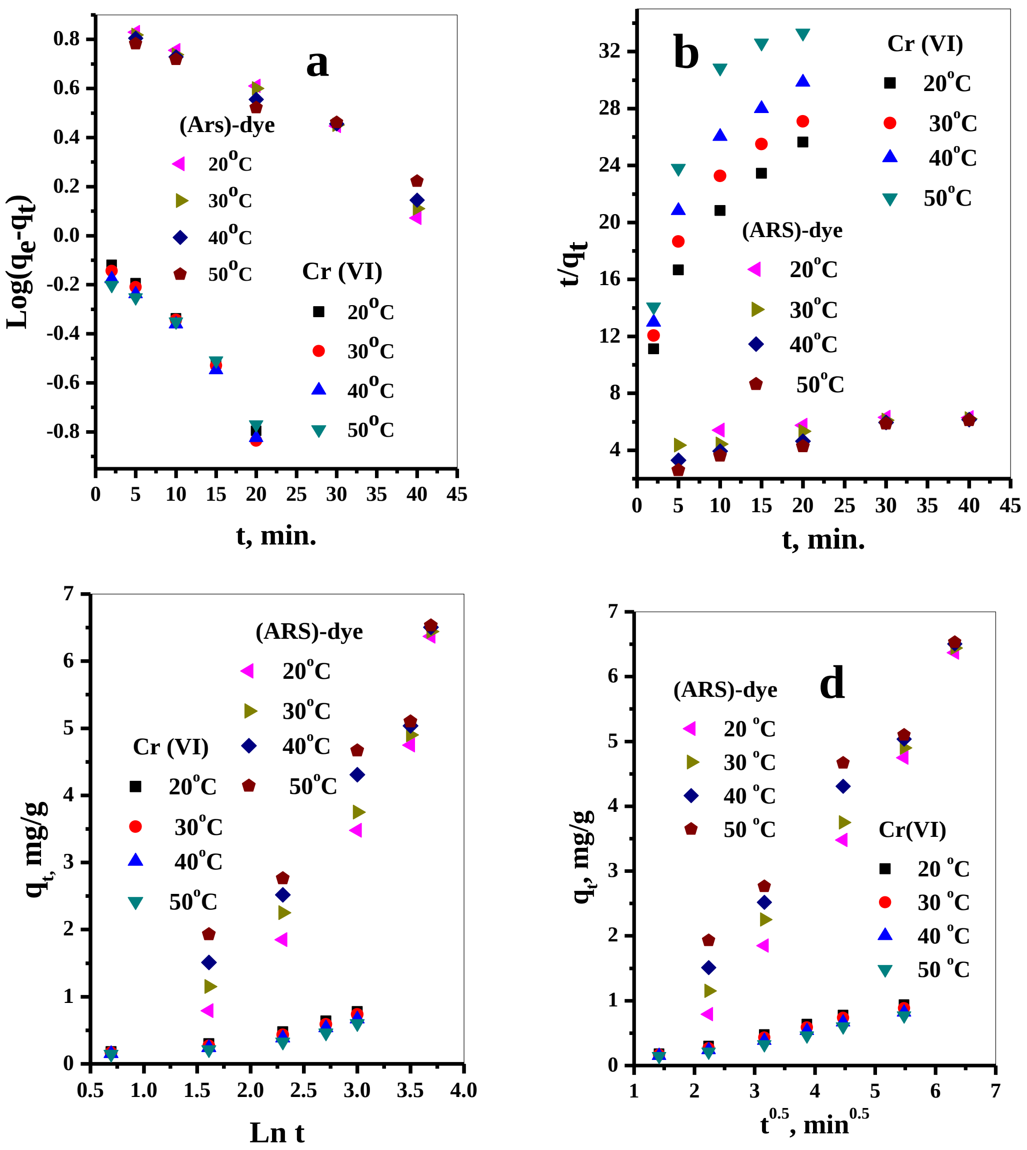


**Figure S1.** The kinetic models of adsorption for adsorption of both Cr(VI) and (ARS)-dye onto 0.05g/10mL $Fe_3O_4$@Talc nanocomposite, (**a**) Pseudo-first order, (**b**) and Pseudo-second order, (**c**) Elovich model, and (**d**) intraparticle diffusion.

([Cr(VI)]= 10 mg/L, pH= 3, T = 20 °C; ([ARS-dye]= 50 mg/L, pH= 2, T = 20 °C).
